# Disentangling Neurodegeneration From Aging in Multiple Sclerosis Using Deep Learning: The Brain-Predicted Disease Duration Gap

**DOI:** 10.1212/WNL.0000000000209976

**Published:** 2024-11-04

**Authors:** Giuseppe Pontillo, Ferran Prados, Jordan Colman, Baris Kanber, Omar Abdel-Mannan, Sarmad Al-Araji, Barbara Bellenberg, Alessia Bianchi, Alvino Bisecco, Wallace J. Brownlee, Arturo Brunetti, Alessandro Cagol, Massimiliano Calabrese, Marco Castellaro, Ronja Christensen, Sirio Cocozza, Elisa Colato, Sara Collorone, Rosa Cortese, Nicola De Stefano, Christian Enzinger, Massimo Filippi, Michael A. Foster, Antonio Gallo, Claudio Gasperini, Gabriel Gonzalez-Escamilla, Cristina Granziera, Sergiu Groppa, Yael Hacohen, Hanne F.F. Harbo, Anna He, Einar A. Hogestol, Jens Kuhle, Sara Llufriu, Carsten Lukas, Eloy Martinez-Heras, Silvia Messina, Marcello Moccia, Suraya Mohamud, Riccardo Nistri, Gro O. Nygaard, Jacqueline Palace, Maria Petracca, Daniela Pinter, Maria A. Rocca, Alex Rovira, Serena Ruggieri, Jaume Sastre-Garriga, Eva M. Strijbis, Ahmed T. Toosy, Tomas Uher, Paola Valsasina, Manuela Vaneckova, Hugo Vrenken, Jed Wingrove, Charmaine Yam, Menno M. Schoonheim, Olga Ciccarelli, James H. Cole, Frederik Barkhof, for the MAGNIMS study group.

**Affiliations:** From the Queen Square Multiple Sclerosis Centre (G.P., F.P., J.C., B.K., O.A.-M., S.A.-A., A. Bianchi, W.J.B., R. Christensen, E.C., S. Collorone, M.A.F., Y.H., A.H., S. Mohamud, R.N., A.T.T., J.W., C.Y., O.C., F.B.), Department of Neuroinflammation, UCL Queen Square Institute of Neurology, University College London, United Kingdom; MS Center Amsterdam (G.P., H.V., F.B.), Radiology and Nuclear Medicine, Vrije Universiteit Amsterdam, Amsterdam Neuroscience, Amsterdam UMC location VUmc, the Netherlands; Departments of Advanced Biomedical Sciences and Electrical Engineering and Information Technology (G.P., A. Brunetti, S. Cocozza), University of Naples “Federico II,” Italy; Centre for Medical Image Computing (F.P., B.K., F.B.), Department of Medical Physics and Biomedical Engineering, University College London, United Kingdom; E-Health Center (F.P.), Universitat Oberta de Catalunya, Barcelona, Spain; Institute of Neuroradiology (B.B., C.L.), St. Josef Hospital, Ruhr-University Bochum, Germany; Department of Advanced Medical and Surgical Sciences (A. Bisecco, A.G.), University of Campania “Luigi Vanvitelli,” Naples, Italy; Translational Imaging in Neurology (ThINK) Basel (A.C., C. Granziera), Department of Biomedical Engineering, Faculty of Medicine, University Hospital Basel, University of Basel; Neurologic Clinic and Policlinic (A.C., C. Granziera, J.K.), MS Center and Research Center for Clinical Neuroimmunology and Neuroscience Basel (RC2NB), University Hospital Basel, University of Basel, Switzerland; Department of Neurosciences, Biomedicine and Movement Sciences (M. Calabrese, M. Castellaro), University of Verona; Department of Information Engineering (M. Castellaro), University of Padova; Department of Medicine, Surgery and Neuroscience (R. Cortese, N.D.S.), University of Siena, Italy; Department of Neurology (C.E., D.P.), Medical University of Graz, Austria; Neuroimaging Research Unit (M.F., M.A.R., P.V.), Division of Neuroscience, IRCCS San Raffaele Scientific Institute, Neurology Unit, Neurorehabilitation Unit, Neurophysiology Service, IRCCS San Raffaele Scientific Institute; Vita-Salute San Raffaele University (M.F., M.A.R., P.V.), Milan; Department of Neurosciences (C. Gasperini, S.R.), San Camillo-Forlanini Hospital, Rome, Italy; Department of Neurology (G.G.-E., S.G.), Focus Program Translational Neuroscience (FTN) and Immunotherapy (FZI), Rhine Main Neuroscience Network (rmn2), University Medical Center of the Johannes Gutenberg University Mainz, Germany; Department of Neurology (H.F.F.H., E.A.H., G.O.N.), Oslo University Hospital; Institute of Clinical Medicine (H.F.F.H., E.A.H., G.O.N.), and Department of Psychology (E.A.H.), University of Oslo, Norway; Neuroimmunology and Multiple Sclerosis Unit Laboratory of Advanced Imaging in Neuroimmunological Diseases (ImaginEM) (S.L., E.M.-H.), Hospital Clinic Barcelona, Fundació de Recerca Clínic Barcelona-Institut d'Investigacions Biomèdiques August Pi i Su, Barcelona, Spain; Department of Neurology (C.L.), St. Josef Hospital, Ruhr-University Bochum, Germany; Nuffield Department of Clinical Neurosciences (S. Messina, J.P.), University of Oxford, United Kingdom; Department of Molecular Medicine and Medical Biotechnology (M.M.), and Department of Neurosciences and Reproductive and Odontostomatological Sciences (M.P.), University of Naples “Federico II”; Department of Human Neurosciences (M.P.), Sapienza University of Rome, Italy; Section of Neuroradiology (A.R.), Department of Radiology, and Centre d'Esclerosi Múltiple de Catalunya (Cemcat) (J.S.-G.), Department of Neurology/Neuroimmunology, Hospital Universitari Vall d'Hebron, Universitat Autònoma de Barcelona, Spain; MS Center Amsterdam (E.M.M.S.), Neurology, Vrije Universiteit Amsterdam, Amsterdam Neuroscience, Amsterdam UMC location VUmc, the Netherlands; Department of Neurology and Center of Clinical Neuroscience (T.U.), and Department of Radiology (M.V.), First Faculty of Medicine, Charles University and General University Hospital, Prague, Czech Republic; MS Center Amsterdam (M.M.S.), Anatomy and Neurosciences, Vrije Universiteit Amsterdam, Amsterdam Neuroscience, Amsterdam UMC location VUmc, the Netherlands; Centre for Medical Image Computing (J.H.C.), Department of Computer Science, and Dementia Research Centre (J.H.C., F.B.), UCL Queen Square Institute of Neurology, University College London, United Kingdom.

## Abstract

**Background and Objectives:**

Disentangling brain aging from disease-related neurodegeneration in patients with multiple sclerosis (PwMS) is increasingly topical. The brain-age paradigm offers a window into this problem but may miss disease-specific effects. In this study, we investigated whether a disease-specific model might complement the brain-age gap (BAG) by capturing aspects unique to MS.

**Methods:**

In this retrospective study, we collected 3D T1-weighted brain MRI scans of PwMS to build (1) a cross-sectional multicentric cohort for age and disease duration (DD) modeling and (2) a longitudinal single-center cohort of patients with early MS as a clinical use case. We trained and evaluated a 3D DenseNet architecture to predict DD from minimally preprocessed images while age predictions were obtained with the DeepBrainNet model. The brain-predicted DD gap (the difference between predicted and actual duration) was proposed as a DD-adjusted global measure of MS-specific brain damage. Model predictions were scrutinized to assess the influence of lesions and brain volumes while the DD gap was biologically and clinically validated within a linear model framework assessing its relationship with BAG and physical disability measured with the Expanded Disability Status Scale (EDSS).

**Results:**

We gathered MRI scans of 4,392 PwMS (69.7% female, age: 42.8 ± 10.6 years, DD: 11.4 ± 9.3 years) from 15 centers while the early MS cohort included 749 sessions from 252 patients (64.7% female, age: 34.5 ± 8.3 years, DD: 0.7 ± 1.2 years). Our model predicted DD better than chance (mean absolute error = 5.63 years, *R*^2^ = 0.34) and was nearly orthogonal to the brain-age model (correlation between DD and BAGs: *r* = 0.06 [0.00–0.13], *p* = 0.07). Predictions were influenced by distributed variations in brain volume and, unlike brain-predicted age, were sensitive to MS lesions (difference between unfilled and filled scans: 0.55 years [0.51–0.59], *p* < 0.001). DD gap significantly explained EDSS changes (*B* = 0.060 [0.038–0.082], *p* < 0.001), adding to BAG (Δ*R*^2^ = 0.012, *p* < 0.001). Longitudinally, increasing DD gap was associated with greater annualized EDSS change (*r* = 0.50 [0.39–0.60], *p* < 0.001), with an incremental contribution in explaining disability worsening compared with changes in BAG alone (Δ*R*^2^ = 0.064, *p* < 0.001).

**Discussion:**

The brain-predicted DD gap is sensitive to MS-related lesions and brain atrophy, adds to the brain-age paradigm in explaining physical disability both cross-sectionally and longitudinally, and may be used as an MS-specific biomarker of disease severity and progression.

## Introduction

In multiple sclerosis, a complex interplay exists between brain aging and disease-related tissue damage accumulation.^1^ Untangling the shared and unique aspects of aging and multiple sclerosis–related neurodegeneration is important to accurately assess disease severity and progression over time and is increasingly relevant as both life expectancy and the average age of patients with multiple sclerosis (PwMS) are increasing.^2^ However, measuring the 2 processes independently is an open challenge because of their substantial overlap and dynamic interaction.^3^

The brain-age paradigm has emerged as a promising data reduction strategy, summarizing complex neuroimaging information into a simple yet clinically relevant biomarker of aging and neurodegeneration.^4^ In brief, machine learning methods are used to model chronological age as a function of brain MRI scans in healthy individuals (HI), and the resulting model of normal brain aging is used for neuroimaging-based age prediction in unseen individuals.^4^ The extent to which an individual deviates from healthy brain aging, expressed as the difference between predicted and chronological age (the brain-age gap [BAG]), has been proposed as an age-adjusted global index of brain health, capturing variations associated with a wide spectrum of neurologic and psychiatric disorders, including multiple sclerosis.^5-7^

However, the BAG metric is designed to be sensitive to those aspects of brain pathology that most resemble healthy aging processes, potentially failing to capture disease-specific effects. Indeed, conceptualizing brain involvement in multiple sclerosis solely as a form of premature/accelerated aging might be reductive since it differs from healthy brain aging not only in grade but also in nature. Brain volume loss, for instance, is known to occur with different spatiotemporal patterns in healthy aging and multiple sclerosis^8^ while white matter lesions are characteristic of multiple sclerosis but do not substantially determine brain-age prediction.^7^ Furthermore, BAG is influenced by early-life genetic and environmental factors, which may not be intrinsically related to aging processes nor to the development of brain pathology.^9^

While there is increasing attention to the prodromal and preclinical aspects of multiple sclerosis, a discrete clinical onset date is almost always identifiable in PwMS which, although inherently ambiguous, might represent an acceptable proxy for disease start and enable the estimation of disease duration (DD).^10^ Of interest, this has been previously used to contextualize individual disease severity in PwMS by referencing a clinical indicator (e.g., the Expanded Disability Status Scale [EDSS] score) to its distribution in patients with comparable DD (i.e., the Multiple Sclerosis Severity Score [MSSS]).^11^

In this study, we proposed a quantitative neuroimaging measure of brain structural damage, assessed through conventional MRI and referenced to DD. We hypothesized that modeling DD in PwMS as a function of structural brain MRI scans would provide a reference standard of multiple sclerosis–related brain damage accumulation. The error associated with the prediction of DD (the brain-predicted DD gap), quantifying the extent to which a patient deviates from the typical disease trajectory, should reflect past and ongoing multiple sclerosis–specific processes and encode biologically and clinically relevant information about disease-related variability. By evaluating it against BAG and longitudinal clinical and MRI data, we aimed to validate the brain-predicted DD gap as a neuroimaging biomarker of multiple sclerosis severity and progression.

Finally, as multiple sclerosis is sometimes theorized as a purely age-dependent disease, with natural history driven by age irrespective of the apparent DD,^12^ we also explored an alternative modeling strategy originating from this alternative conceptualization of the disease. Specifically, we modeled chronological age in PwMS to estimate a reference trajectory of multiple sclerosis–specific brain aging (MS-age) and tested the corresponding prediction error (the brain-predicted MS-age gap) as a biomarker of disease severity and progression.

## Methods

### Participants

In this retrospective multicentric study, we collected MRI and clinicodemographic data of patients diagnosed with multiple sclerosis according to the 2010 McDonald criteria^13^ or clinically isolated syndrome.^10^ Exclusion criteria were age younger than 15 or older than 75 years and the presence of other relevant neurologic, psychiatric, or systemic conditions.

We gathered 3D T1-weighted (T1w) brain MRI scans of patients from multiple European MS centers for modeling age and DD. We considered only 1 MRI scan per patient for the analyses, selecting the first time point when multiple scans were available. A T2-weighted fluid-attenuated inversion recovery (FLAIR) scan was also required for all patients of the age and DD modeling cohort to build FLAIR-based models and automatically segment T2-hyperintense lesions for interpretability analyses. For further validation of the age and DD models, we used 3D T1w brain images of a single-center longitudinal cohort of patients with a first scan in the early phases of the disease (<5 years from clinical onset) as a clinical use case. Physical disability was scored using EDSS at the time of MRI.

### Standard Protocol Approvals, Registrations, and Patient Consents

Written informed consent was obtained from each patient independently at each center following standard procedures, including parental consent for minor patients. The final protocol for this study was reviewed and approved by the local ethics committees and the MAGNIMS Study Group Steering Committee (magnims.eu).

### Deep Learning Age and DD Modeling

**Figure 1:**
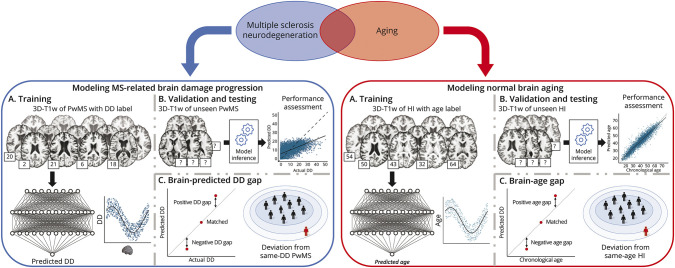
Conceptual Framework of the Study With the brain-age paradigm, chronological age is modeled as a function of brain MRI scans in healthy individuals and the resulting model of normal brain aging is used for neuroimaging-based age prediction in unseen individuals. The extent to which an individual deviates from healthy brain aging, expressed as the difference between predicted and chronological age (the brain-age gap), is an age-adjusted global index of brain health. We proposed to complement this approach by further modeling DD in PwMS as a function of brain MRI scans, to provide a reference standard of multiple sclerosis–related brain damage accumulation. The error associated with the prediction of DD (the brain-predicted DD gap), quantifying the extent to which a patient deviates from the typical disease trajectory, is a DD-adjusted global measure of multiple sclerosis–specific brain damage. DD = disease duration; HI = healthy individuals; MS = multiple sclerosis; PwMS = patients with MS; T1w = T1-weighted.

A schematic illustration of the conceptual design of the study is shown in Figure 1.

T1w scans were used as input for age and DD prediction models. Minimal preprocessing was performed with ANTsPyNet^14^ and included N4 bias field correction, skull stripping, and affine registration to the 1-mm isotropic MNI152 template.

For the prediction of age and the estimation of BAG, we used the DeepBrainNet algorithm, an external model based on a 2-dimensional convolutional neural network architecture that has been extensively validated.^15^ The BAG model was considered as a benchmark in subsequent analyses.

For the prediction of DD, an in-house model was trained, validated, and tested on the age and DD modeling cohort, which was randomly split into training (n = 2,811, 64%), validation (n = 703, 16%), and test (n = 878, 20%) sets. Our model was built on the 3D DenseNet264 architecture, adapted from the implementation available at Project MONAI^16^ by adding a linear regression layer for the prediction of a continuous variable and a 0.2 dropout rate after each dense layer to reduce overfitting. The DenseNet architecture was chosen because it can be considered a leading-edge architecture for computer vision tasks,^17^ has already demonstrated high performance in predicting brain age,^15,18^ and is readily available as an “off-the-shelf” network within the project MONAI framework, ensuring ease of use and reproducibility. Before being presented to the model, images were resampled to 1.5-mm^3^ voxels to reduce array size and computational burden while retaining anatomical details and online data augmentation was performed, including random spatial and intensity transformations, to make the network further invariant to image quality variations and site effects. A log(x + 1) transformation was applied to the outcome variable to account for the highly positively skewed distribution of DD values. Mean absolute error (MAE) and coefficient of determination (*R*^2^) were used to quantify model performance. The brain-predicted DD gap was computed as the difference between predicted and actual DD values. Modeling was performed with PyTorch 1.12.0^19^ using 1 NVIDIA Tesla T4 16 GB graphics processing unit. The performance in predicting DD in the test set was also compared with that of simpler linear models based on volumes of lesions and gray matter regions obtained by automatically segmenting T1w and FLAIR scans using SAMSEG^20^ and FastSurfer,^21^ respectively. To model multiple sclerosis–specific brain aging, we trained, validated, and tested the same network architecture to predict chronological age in the age and DD modeling cohort. The brain-predicted MS-age gap was computed as the difference between predicted and chronological age. A conceptual outline of the MS-age modeling strategy is shown in eFigure 1.

As high-resolution T1w sequences are not mandatory for the diagnosis or monitoring of MS according to current guidelines, unlike T2-FLAIR which is considered to be the core sequence,^22^ we also built and evaluated similar models predicting DD and MS-age based only on FLAIR scans in the age and DD modeling cohort.

### Model Interpretability

To scrutinize model predictions on the test set, we used guided backpropagation to obtain saliency maps highlighting regions of the input image that are most influential for the model's predictions.^23^

Furthermore, to better understand the imaging patterns underlying the predictions, we conducted a correlation analysis in the test set between age and DD gaps and volumes of lesions and gray matter regions, while correcting for age, age^2^, DD, sex, and estimated total intracranial volume.

Finally, we investigated the impact of MS lesions on age and DD predictions: lesions were artificially removed from T1w images of the test set using FSL lesion-filling algorithm,^24^ both “lesion-filled” and “unfilled” scans were run through the prediction procedures, and resulting values were compared with paired sample *t* tests and Bland-Altman plots.

### Statistical Analysis

Statistical analyses were performed using R (version 4.1.2), with a statistical significance level set at *p* < 0.05. Cross-sectional associations between age and DD gaps and EDSS scores were investigated in the test set using linear models including also age, age^2^ (to account for the nonlinear effect of age), DD, and sex. To assess the additional values of the DD and MS-age gap metrics over the “classical” BAG in explaining EDSS variance, the corresponding models were compared using *F* tests.

In the early multiple sclerosis cohort, the longitudinal evolutions of EDSS change, BAG, DD, and MS-age gaps were analyzed using a multilevel linear model framework, with time points nested within patients and random intercept and slope of follow-up time per patient, also including the fixed effects of age, age^2^, and sex. When modeling the DD gap, the fixed effect of DD was also included in the model to correct for DD-related bias (i.e., the underestimation of DD in long-standing PwMS, and vice versa). From these growth models, individualized changes per year (i.e., annualized) were extracted as the individual-level coefficients of the follow-up time term, corresponding to the sum of the fixed and random effects. Then, we explored how longitudinal changes in brain MRI-derived measures related to changes in physical disability (i.e., EDSS changes) by correlating the corresponding annualized changes in patients with at least 2 visits (n = 200). Similar to the cross-sectional analysis, the value of adding the longitudinal evolution of DD or MS-age gap to BAG change over time for explaining EDSS worsening was assessed by comparing the corresponding models with *F* tests.

### Data Availability

Data from patients are controlled by the respective centers (listed in Table 1) and are, therefore, not publicly available. Request to access the data should be forwarded to data controllers through the corresponding author. The trained DD and MS-age models will be made available at github.com/giupontillo.

**Table 1:** Demographic and Clinical Characteristics of the Studied Population

Cohort	N	Female sex, %	Age, y			DD, y			EDSS			CIS/RR/SP/PP/NA
			Mean	SD	Range	Mean	SD	Range	N	Median	Range	
Age and DD modeling	4,392	69.7	42.8	10.6	16.4–72.8	11.4	9.3	0.0–51.6	4,341	2.0	0.0–7.5	330/3,340/537/170/15
Barcelona I	885	69.6	44.3	9.9	18.4–70.6	13.5	8.7	0.0–45.8	878	2.0	0.0–6.5	196/395/190/89/15
Barcelona II	61	70.5	48.9	10.4	25.6–72.2	19.7	9.5	8.4–46.1	61	2.5	1.0–6.5	0/52/9/0/0
Basel	100	55.0	46.3	13.6	18.2–71.9	14.6	12.0	0.5–47.9	100	3.0	0.0–7.0	1/73/18/8/0
Bochum	101	66.3	34.2	10.7	18.4–59.5	0.7	0.7	0.0–2.5	101	1.5	0.0–5.0	52/49/0/0/0
Graz	143	60.8	40.3	10.0	20.1–69.0	10.6	8.3	0.4–39.1	143	1.0	0.0–7.0	3/127/10/3/0
Mainz	350	69.4	34.8	10.5	17.0–69.0	2.8	4.6	0.0–33.0	347	1.0	0.0–6.5	64/285/0/1/0
Milan	64	57.8	43.6	10.5	22.5–62.9	11.7	10.3	0.0–39.0	64	4.5	1.0–7.5	0/30/27/7/0
Naples I	220	65.0	40.0	12.6	16.4–72.3	10.4	8.5	0.0–39.4	220	3.5	0.0–7.5	2/158/36/24/0
Naples II	63	61.9	37.9	10.8	20.9–61.7	9.0	8.4	0.1–32.3	63	2.0	0.0–6.5	1/52/5/5/0
Oslo	401	70.8	38.5	10.2	18.5–68.3	4.7	6.2	0.0–36.9	372	2.0	0.0–7.0	7/377/11/6/0
Oxford	16	56.2	44.8	6.5	31.9–56.0	10.8	4.7	1.8–19.5	16	2.0	0.0–6.0	0/16/0/0/0
Prague	1,785	72.4	45.4	8.9	20.1–72.8	14.0	8.9	0.0–51.6	1,774	2.5	0.0–7.5	0/1,540/226/19/0
Rome	105	72.4	43.1	11.3	17.7–65.3	10.7	8.7	0.7–36.9	105	2.0	0.0–6.5	1/99/3/2/0
Siena	47	72.3	44.0	12.0	17.9–71.3	12.7	8.4	1.0–38.4	47	1.5	0.0–6.5	0/42/0/5/0
Verona	51	72.5	40.0	11.4	20.3–65.5	9.1	8.7	0.0–34.9	50	2.0	0.0–7.0	3/45/2/1/0
Early multiple sclerosis	252	64.7	34.5	8.3	19.4–64.5	0.7	1.2	0.0–4.5	231	1.0	0.0–6.5	110/142/0/0/0
London I	160	64.4	35.2	8.8	19.4–64.5	1.0	1.5	0.0–4.5	150	1.0	0.0–6.5	49/111/0/0/0
London II	92	65.2	33.1	7.2	19.9–53.7	0.2	0.3	0.0–3.1	81	1.0	0.0–3.5	61/31/0/0/0

Abbreviations: CIS = clinically isolated syndrome; DD = disease duration; NA = not available; PP = primary progressive; RR = relapsing remitting; SP = secondary progressive.

## Results

### Participants

MRI scans of 4,392 unique PwMS from 15 European MS centers were collected for the age and DD modeling cohort, acquired between 2011 and 2022. For the 1,049 patients for whom more than 1 MRI scan was available (mean follow-up time = 2.3 years, range: 0.2–4.4), the first one was used for subsequent analyses. The early multiple sclerosis longitudinal validation cohort was composed of 252 patients and 749 sessions, acquired between 1999 and 2015, with a mean follow-up time of 4.5 years (range: 0.2–19.1). Demographic and clinical characteristics of the studied population are given in Table 1 while the details of the different acquisition protocols are provided in eTable 1.

### Age and DD Models

**Figure 2:**
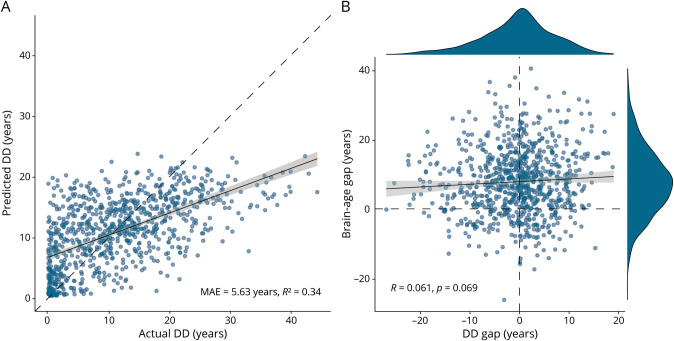
Modeling Disease Duration in Patients With Multiple Sclerosis (A) Scatterplot showing the relationship between the actual disease duration values in the test set (N = 878) and the ones predicted by the model. (B) Scatterplot showing the relationship between the disease duration gap and the brain-age gap (obtained with the DeepBrainNet model) in the test set; marginal density plots are also shown, portraying the distribution of the 2 variables. Linear fit lines are shown as solid lines (with corresponding 95% confidence intervals in gray) while dashed lines represent the line of identity (A) and horizontal and vertical zero reference lines (B), respectively. DD = disease duration; MAE = mean absolute error.

The DeepBrainNet model predictions on the test set showed that multiple sclerosis was associated with older appearing brains (mean BAG: 7.81 years, 95% CI 7.19–8.43). As for the prediction of DD, the out-of-sample performance of the model was well above chance level (test set MAE = 5.63 years, *R*^2^ = 0.34) (Figure 2A). To help contextualize the performance of the DD prediction model, we computed *R*^2^ values for the association of DD with established MRI biomarkers in the test set for comparison: total lesion volume (*R*^2^ = 0.10), thalamic volume (*R*^2^ = 0.14), and the combination of lesion burden and 100 regional volumes from FastSurfer's segmentation (*R*^2^ = 0.28). As for the MS-age model, predictions on the test set were highly accurate (MAE = 3.78 years, *R*^2^ = 0.80) (eFigure 2A).

When looking at the relationship between these metrics, DD gap and BAG values were nearly orthogonal to each other (*r* = 0.06, 95% CI 0.00–0.13, *p* = 0.07), suggesting that the models' predictions were largely independent (Figure 2B). On the contrary, MS-age gap still moderately correlated with BAG (*r* = 0.35, 95% CI 0.29–0.40, *p* < 0.001), revealing a higher degree of entanglement between the 2 models (eFigure 2B).

FLAIR-based models yielded comparable out-of-sample accuracies for the prediction of DD (MAE = 5.70 years, *R*^2^ = 0.33) and MS-age (MAE = 3.26 years, *R*^2^ = 0.85) (eFigure 3), with FLAIR-based and T1w-based predictions being highly correlated (*r* = 0.79, 95% CI 0.76–0.81, for DD, and *r* = 0.91, 95% CI 0.90–0.92, for age) (eFigure 4).

**Figure 3:**
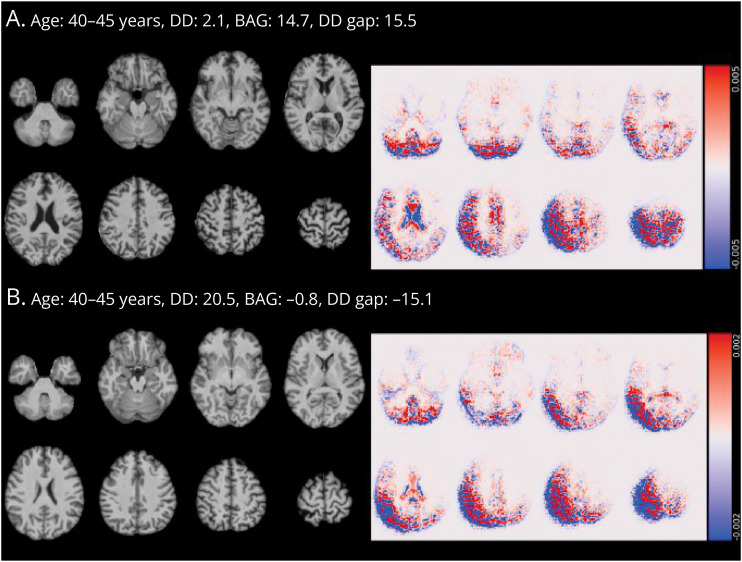
Guided Backpropagation Analysis to Interrogate Brain Regions Influencing the Model for the Prediction of Disease Duration Lightbox view of selected slices from the quasi-raw T1w volumes (on the left) and corresponding guided backpropagation–derived saliency maps (on the right) of 2 representative PwMS exhibiting extremely positive (A) or negative (B) values of the DD gap. For saliency maps, both positive (positively correlated with the output, in red) and negative (negatively correlated with the outcome, in blue) magnitudes are shown. In both cases, the model focuses mostly on regions that seem to be related to (the widening of) the CSF spaces. BAG = brain-age gap; DD = disease duration; PwMS = patients with multiple sclerosis; T1w = T1-weighted.

**Figure 4:**
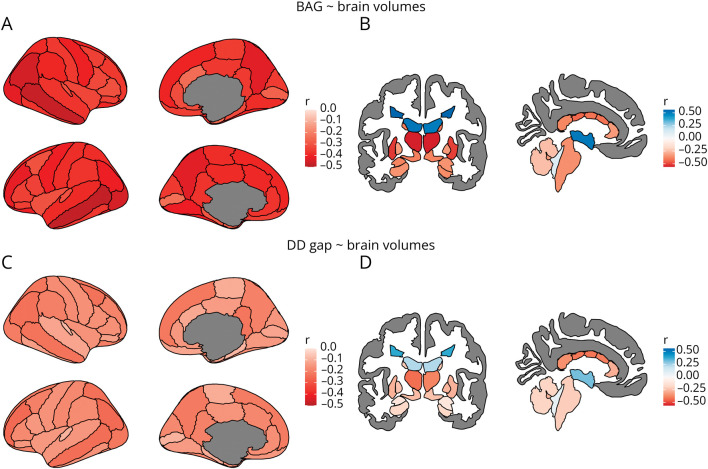
Correlations Between Brain-Age and Disease Duration Gaps and Regional Brain and Lesion Volumes In the upper row, plots show the correlations between brain-age gap values and cortical (A) and subcortical/lesion (B) volumes. In the bottom row, plots show the correlations between disease duration gap values and cortical (C) and subcortical/lesion (D) volumes. Shown are the Pearson correlation coefficients resulting from partial correlation analyses correcting for age, age^2^, disease duration, sex, and estimated total intracranial volume. The cortex is parcellated according to the DKT atlas.^21^ BAG = brain-age gap; DD = disease duration.

**Figure 5:**
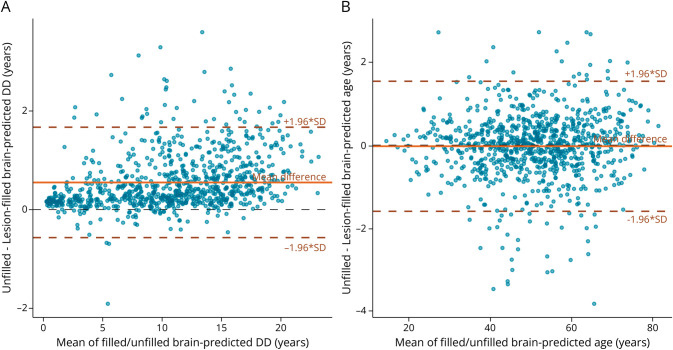
Impact of MS Lesions on Age and DD Predictions Bland-Altman plot of brain-predicted DD (A) and age (B) from unfilled and filled T1w scans. The plots show the mean value from the 2 measures for each patient (x-axis) and the difference between the 2 measures (y-axis). The mean difference lines are solid, and the corresponding limits of agreement (±1.96 × SD of difference) are dashed lines. DD = disease duration; MS = multiple sclerosis.

The interpretability analysis showed that both the DD and MS-age models focused on regions that seem to be primarily related to (the widening of) the CSF spaces (Figure 3 and eFigure 5). In addition, all age and DD gap measures correlated diffusely with regional brain atrophy and lesion burden, with the greatest effect sizes observed for BAG values (and the lowest for the MS-age gap) and no clear anatomical specificity (Figure 4 and eFigure 6). As for the impact of MS lesions, there was a significant impact of the filling procedure on brain-predicted DD values (mean difference between unfilled and filled scans: 0.55 years, 95% CI 0.51–0.59, *p* < 0.001) (Figure 5A), with no evident systematic bias caused by lesion filling for brain-age predictions (mean difference: −0.03 years, 95% CI −0.08 to 0.03, *p* = 0.31) (Figure 5B). The MS-age model was also slightly sensitive to the filling procedure (mean difference: 0.07 years, 95% CI 0.04–0.10, *p* < 0.001) (eFigure 7).

### Brain-Age and DD Gaps Independently Explain Physical Disability

**Figure 6:**
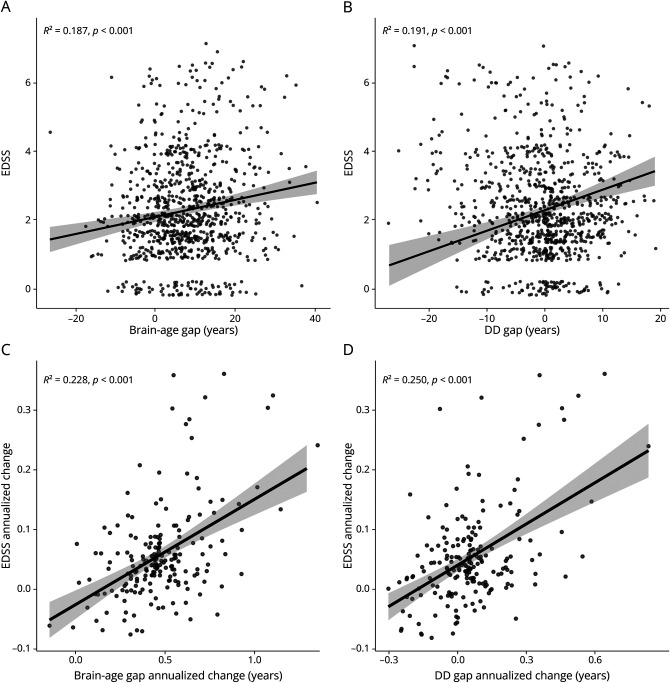
Relationships Between Brain-Age and Disease Duration Gaps and Physical Disability In the upper row, scatterplots show the marginal effects on EDSS scores of the brain-age (A) and disease duration (B) gap metrics (regression models were corrected for the effects of age, age^2^, disease duration, and sex). In the bottom row, scatterplots show the relationship between annualized changes in EDSS and brain-age (C) and disease duration (D) gaps. Linear fit lines are shown as solid lines (with corresponding 95% confidence intervals in gray). DD = disease duration; EDSS = Expanded Disability Status Scale.

In the test set, both BAG (*B* = 0.026, 95% CI 0.016–0.036, β = 0.160, *p* < 0.001) and DD gap (*B* = 0.060, 95% CI 0.038–0.082, β = 0.305, *p* < 0.001) were positively associated with EDSS scores (Figure 6, A and B and eTable 2). A positive association was also found for the MS-age gap metric (*B* = 0.031, 95% CI 0.011–0.051, β = 0.099, *p* < 0.001) (eFigure 8 and eTable 3).

When investigating the incremental value of multiple sclerosis–specific metrics in explaining EDSS change in addition to BAG, the DD gap significantly added to the baseline model (Δ*R*^2^ = 0.012, *p* < 0.001) (eTable 2) while the inclusion of the MS-age gap did not significantly improve the model fit (Δ*R*^2^ = 0.002, *p* = 0.10) (eTable 3).

With FLAIR-based predictions, the DD gap metric was still positively related to the EDSS score (*B* = 0.040, 95% CI 0.022–0.058, β = 0.160, *p* < 0.001) while the association of MS-age gap with disability was nonsignificant (*B* = 0.005, 95% CI −0.017 to 0.027, β = 0.015, *p* = 0.66) (eFigure 9 and eTable 4).

### Longitudinal Brain-Age and DD Gap Increases Independently Explain EDSS Worsening

In the early multiple sclerosis cohort, growth models revealed significant EDSS worsening (*B* = 0.058, 95% CI 0.027–0.089, *p* < 0.001) and BAG increase (*B* = 0.472, 95% CI 0.298–0.646, *p* < 0.001) over time. Both DD (*B* = 0.057, 95% CI −0.033 to 0.147, *p* = 0.22) and MS-age (*B* = 0.016, 95% CI −0.086 to 0.118, *p* = 0.76) gaps only exhibited a slight, nonsignificant, upward trend (eFigure 10 and eTable 5).

The annualized change in the EDSS score correlated with annualized changes in both BAG (*r* = 0.48, 95% CI 0.36–0.58, *p* < 0.001) and DD gap (*r* = 0.50, 95% CI 0.39–0.60, *p* < 0.001) (Figure 6, C and D and eTable 6), whereas the correlation between annualized changes in EDSS scores and MS-age gap did not reach statistical significance (*r* = 0.11, 95% CI −0.03 to 0.25, *p* = 0.12) (eFigure 11 and eTable 7).

When assessing how longitudinal changes in multiple sclerosis–specific metrics contributed to explaining EDSS worsening, the DD gap significantly added to the baseline model including BAG change over time (Δ*R*^2^ = 0.064, *p* < 0.001) (eTable 6) while the addition of the MS-age gap metric did not significantly improve the model fit (Δ*R*^2^ = 0.010, *p* = 0.13) (eTable 7).

## Discussion

Using deep learning, we separately modeled aging and disease-specific effects from structural brain MRI scans of a large multicentric sample of PwMS. We validated the DD gap as a biologically and clinically meaningful global measure of multiple sclerosis–specific brain damage, adding to the information provided by models of healthy brain aging as a biomarker of disease severity and progression.

While age is often treated as a mere confounder in neuroimaging analyses, brain aging and multiple sclerosis are intimately intertwined. On the one hand, the relationship between age and the brain is shaped by the disease and encodes disease-related information. On the other hand, age is an essential modifier of multiple sclerosis clinical course and treatment response.^25,26^ Understanding the complex interaction between aging and neurodegeneration and disentangling the overlapping and distinct mechanisms underlying the 2 processes bears significant transdiagnostic relevance and is the topic of increasing research interest.^27,28^

The brain-age paradigm offers a window into this problem and has been previously used to characterize neurodegeneration in multiple sclerosis because of its sensitivity to brain aging–like patterns.^5-7,29,30^ In line with previous studies, our results confirmed that, when observed through the lens of healthy aging, the brains of PwMS look older than normal (around 8 years on average), suggesting that at least some of the disease-related variance in brain structure can be effectively modeled as premature/accelerated brain aging.

When trying to disentangle disease-specific effects, the proposed DD gap metric exhibited a low correlation with BAG, supporting the relative independence between the 2 measures and the underlying phenomena. It should be noted that DD is an intrinsically noisy measure, relying on the date of clinical onset, which is often assigned retrospectively based on the subjective recollection of symptoms. Nevertheless, the model performance was above chance level and explained considerably more variance in DD than other established measures of multiple sclerosis–related brain involvement such as total lesion and thalamic volumes. On the contrary, the MS-age model was highly accurate, nearly approaching the performance of state-of-the-art deep learning models of healthy brain aging,^15,18,31,32^ but the corresponding gap measure was highly correlated with BAG, suggesting a greater degree of residual dependence from healthy brain aging patterns. Of interest, highly similar predictions were obtained with models relying on FLAIR scans, which are more commonly available in PwMS, supporting the feasibility of the proposed approach in “real-world” clinical settings.

The interpretability analyses showed that all measures were influenced by regional brain volumes, with the lowest effect sizes observed for the MS-age gap metric, revealing the lower capacity of the corresponding model to capture interindividual variability. In line with what has been previously observed for healthy brain aging models,^33,34^ age and DD predictions were influenced by spatially distributed, rather than localized, variations in brain volume. Of interest, the presence of lesions did not directly influence BAG values while MS-age and, most prominently, DD predictions on unfilled scans were systematically higher than those obtained on lesion-filled counterparts, suggesting that multiple sclerosis–specific models can effectively measure disease-related phenomena that are not captured by the classical brain-age paradigm.

This idea was further supported by the association with physical disability, with the DD gap metric explaining additional variance in the EDSS score compared with BAG alone. While the explanatory effect of the DD gap regarding clinical disability and its added value over BAG were relatively small, these results should be contextualized in light of the high level of nondisease-related interindividual variability present in “real-world,” multicentric data sets like ours. In addition, the clinimetric weaknesses of EDSS should also be considered because it is strongly biased toward motor/ambulatory capacity, which is heavily influenced by spinal cord damage (not assessed in our work).^35^

Longitudinal trajectories estimated on the early multiple sclerosis cohort substantiated the biological interpretation of the investigated metrics. EDSS score, as a measure of physical disability, and BAG, expressing the deviation from healthy brain aging, tend to increase over time on average as a reflection of disease progression. Conversely, multiple sclerosis–specific metrics express deviation from the average PwMS and, therefore, do not exhibit significant group-level change over time.

It should be noted that early multiple sclerosis represents the ideal setting to analytically separate aging and disease-specific effects as the relative contribution of normal aging to brain atrophy is low and the amount of disease-related variance in brain structure that is not explained by aging is higher.^8^ Indeed, a ceiling effect is observable for brain structural damage, with the trajectories of brain volume change in PwMS and HI tending to align in the older patients.^36^

From the clinical perspective, the longitudinal association between BAG and physical disability has been previously demonstrated.^7^ We showed that disability worsening is also paralleled by the increase in DD gap, reflecting accelerated progression of multiple sclerosis–specific brain damage compared with reference trajectories and adding to the BAG metric in explaining EDSS change over time. Of interest, in the longitudinal analyses, with the within-individual design reducing the variability/noise in the data and enhancing sensitivity, the magnitude of the observed explanatory effects regarding clinical disability was substantially greater, confirming that age and DD gaps are more powerful as disease biomarkers in the longitudinal setting.^7^

Taken together, our results show that complementing the brain-age paradigm with models explicitly designed to capture disease-specific effects allows us to comprehensively measure both aging-like and nonaging-like aspects of brain pathology, providing a more accurate explanation of brain damage and related disability in PwMS. The DD gap metric, in particular, is sensitive to imaging patterns (and underlying biological processes) that are relatively independent from healthy brain aging and, therefore, adds to classical brain-age models as a disease-specific biomarker. The model of multiple sclerosis–specific aging, on the contrary, is not as specific, probably because of its sensitivity to physiologic, nondisease-related variability across individuals, and does not seem to add to the classical brain-age paradigm.

Our study is not without limitations. First, the DD model is prone to biases. As mentioned, DD is an intrinsically noisy measure, with the length of the preclinical phase being influenced by several, not necessarily random, factors.^37^ For a more accurate estimation of reference disease trajectories, we need more accurate estimates of disease onset relying on objective biomarkers.^37,38^ In addition, consideration is needed on the potential confounding role of disease-modifying drugs. In recent years, the therapeutic landscape in multiple sclerosis has drastically changed, with the proliferation of highly effective treatment options making PwMS with a more recent diagnosis more likely to have milder disease courses. More complex models taking into account the effect of treatment as a disease course modifier will be needed to solve this possible bias. Furthermore, some caution is needed when interpreting gap values because of their DD dependence (i.e., the underestimation of DD in PwMS with longer disease, and vice versa), which we accounted for by adjusting statistical analyses for age and DD. Similar to healthy brain aging models, care should be taken to apply statistical bias correction before or during downstream analyses,^39^ and further work is warranted to determine the best way to solve this fundamental defect.^40^ It is also important to acknowledge that brain-predicted estimates of age and DD, such as brain volumetric quantifications, may be influenced by factors such as scanner/sequence characteristics or brain volume changes due to physiologic diurnal fluctuations, hydration status, or medications. To fully understand and quantify the impact of these confounders on the proposed metrics and to determine the appropriateness of different harmonization solutions, technical validation studies are necessary. Similarly, further clinical validation with additional outcome measures, longer follow-up time, and more varied, real-world, clinical populations will be needed to fully assess the potential of the DD gap metric for patient stratification in clinical practice. In addition, as the average age of PwMS increases, the influence of comorbidities on MS clinical course becomes increasingly relevant, and studies with detailed multisystem clinical annotations will be necessary to assess their impact on the proposed metrics.^41^ In addition, for the proposed models to be useful in a real-world setting, their confidence in a prediction must also be known, warranting future studies using uncertainty quantification methods to estimate the trustworthiness of healthy brain aging and disease-specific models.^42^ It is also worth noting that the realm of deep learning methods offers possible alternatives to solve the problem of unraveling brain aging and disease-specific effects, with disentangled representation learning approaches being particularly promising in this regard and potentially representing a crucial area for future research.^43,44^ Finally, while we only relied on structural MRI for our models to align with the existing literature on brain-age prediction, other contrasts may also convey relevant information and/or be more accessible in clinical practice, warranting future studies using additional MRI modalities, alone or in combination.

In conclusion, we demonstrated that the DD gap is a clinically meaningful measure of multiple sclerosis–specific brain damage, adding to models of healthy brain aging. By condensing the complex information contained in routinely acquired brain MRI scans into a simple and intuitive biomarker of disease severity and progression, it may represent a powerful tool for the stratification of PwMS in both clinical and research settings.

## Appendix 1 Authors

**Table:**

Name	Location	Contribution
Giuseppe Pontillo, MD, PhD	Queen Square Multiple Sclerosis Centre, Department of Neuroinflammation, UCL Queen Square Institute of Neurology, University College London, United Kingdom; MS Center Amsterdam, Radiology and Nuclear Medicine, Vrije Universiteit Amsterdam, Amsterdam Neuroscience, Amsterdam UMC location VUmc, the Netherlands; Departments of Advanced Biomedical Sciences and Electrical Engineering and Information Technology, University of Naples “Federico II,” Italy	Drafting/revision of the manuscript for content, including medical writing for content; major role in the acquisition of data; study concept or design; analysis or interpretation of data
Ferran Prados, PhD	Queen Square Multiple Sclerosis Centre, Department of Neuroinflammation, UCL Queen Square Institute of Neurology, and Centre for Medical Image Computing, Department of Medical Physics and Biomedical Engineering, University College London, United Kingdom; E-Health Center, Universitat Oberta de Catalunya, Barcelona, Spain	Drafting/revision of the manuscript for content, including medical writing for content; analysis or interpretation of data
Jordan Colman, MD	Queen Square Multiple Sclerosis Centre, Department of Neuroinflammation, UCL Queen Square Institute of Neurology, University College London, United Kingdom	Drafting/revision of the manuscript for content, including medical writing for content; analysis or interpretation of data
Baris Kanber, PhD	Queen Square Multiple Sclerosis Centre, Department of Neuroinflammation, UCL Queen Square Institute of Neurology, and Centre for Medical Image Computing, Department of Medical Physics and Biomedical Engineering, University College London, United Kingdom	Drafting/revision of the manuscript for content, including medical writing for content; analysis or interpretation of data
Omar Abdel-Mannan, MD	Queen Square Multiple Sclerosis Centre, Department of Neuroinflammation, UCL Queen Square Institute of Neurology, University College London, United Kingdom	Drafting/revision of the manuscript for content, including medical writing for content; major role in the acquisition of data
Sarmad Al-Araji, MD	Queen Square Multiple Sclerosis Centre, Department of Neuroinflammation, UCL Queen Square Institute of Neurology, University College London, United Kingdom	Drafting/revision of the manuscript for content, including medical writing for content; major role in the acquisition of data
Barbara Bellenberg, PhD	Institute of Neuroradiology, St. Josef Hospital, Ruhr-University Bochum, Germany	Drafting/revision of the manuscript for content, including medical writing for content; major role in the acquisition of data
Alessia Bianchi, MD, PhD	Queen Square Multiple Sclerosis Centre, Department of Neuroinflammation, UCL Queen Square Institute of Neurology, University College London, United Kingdom	Drafting/revision of the manuscript for content, including medical writing for content; major role in the acquisition of data
Alvino Bisecco, MD, PhD	Department of Advanced Medical and Surgical Sciences, University of Campania “Luigi Vanvitelli,” Naples, Italy	Drafting/revision of the manuscript for content, including medical writing for content; major role in the acquisition of data
Wallace J. Brownlee, MD, PhD	Queen Square Multiple Sclerosis Centre, Department of Neuroinflammation, UCL Queen Square Institute of Neurology, University College London, United Kingdom	Drafting/revision of the manuscript for content, including medical writing for content; major role in the acquisition of data
Arturo Brunetti, MD	Departments of Advanced Biomedical Sciences and Electrical Engineering and Information Technology, University of Naples “Federico II,” Italy	Drafting/revision of the manuscript for content, including medical writing for content; major role in the acquisition of data
Alessandro Cagol, MD	Translational Imaging in Neurology (ThINK) Basel, Department of Biomedical Engineering, Faculty of Medicine, and Neurologic Clinic and Policlinic, MS Center and Research Center for Clinical Neuroimmunology and Neuroscience Basel (RC2NB), University Hospital Basel, University of Basel, Switzerland	Drafting/revision of the manuscript for content, including medical writing for content; major role in the acquisition of data
Massimiliano Calabrese, MD	Department of Neurosciences, Biomedicine and Movement Sciences, University of Verona, Italy	Drafting/revision of the manuscript for content, including medical writing for content; major role in the acquisition of data
Marco Castellaro, PhD	Department of Neurosciences, Biomedicine and Movement Sciences, University of Verona; Department of Information Engineering, University of Padova, Italy	Drafting/revision of the manuscript for content, including medical writing for content; major role in the acquisition of data
Ronja Christensen, MD	Queen Square Multiple Sclerosis Centre, Department of Neuroinflammation, UCL Queen Square Institute of Neurology, University College London, United Kingdom	Drafting/revision of the manuscript for content, including medical writing for content; major role in the acquisition of data
Sirio Cocozza, MD, PhD	Departments of Advanced Biomedical Sciences and Electrical Engineering and Information Technology, University of Naples “Federico II,” Italy	Drafting/revision of the manuscript for content, including medical writing for content; major role in the acquisition of data
Elisa Colato, PhD	Queen Square Multiple Sclerosis Centre, Department of Neuroinflammation, UCL Queen Square Institute of Neurology, University College London, United Kingdom	Drafting/revision of the manuscript for content, including medical writing for content; major role in the acquisition of data
Sara Collorone, MD, PhD	Queen Square Multiple Sclerosis Centre, Department of Neuroinflammation, UCL Queen Square Institute of Neurology, University College London, United Kingdom	Drafting/revision of the manuscript for content, including medical writing for content; major role in the acquisition of data
Rosa Cortese, MD, PhD	Department of Medicine, Surgery and Neuroscience, University of Siena, Italy	Drafting/revision of the manuscript for content, including medical writing for content; major role in the acquisition of data
Nicola De Stefano, MD, PhD	Department of Medicine, Surgery and Neuroscience, University of Siena, Italy	Drafting/revision of the manuscript for content, including medical writing for content; major role in the acquisition of data
Christian Enzinger, MD, PhD	Department of Neurology, Medical University of Graz, Austria	Drafting/revision of the manuscript for content, including medical writing for content; major role in the acquisition of data
Massimo Filippi, MD, PhD	Neuroimaging Research Unit, Division of Neuroscience, IRCCS San Raffaele Scientific Institute, Neurology Unit, Neurorehabilitation Unit, Neurophysiology Service, IRCCS San Raffaele Scientific Institute; Vita-Salute San Raffaele University, Milan, Italy	Drafting/revision of the manuscript for content, including medical writing for content; major role in the acquisition of data
Michael A. Foster, MD	Queen Square Multiple Sclerosis Centre, Department of Neuroinflammation, UCL Queen Square Institute of Neurology, University College London, United Kingdom	Drafting/revision of the manuscript for content, including medical writing for content; major role in the acquisition of data
Antonio Gallo, MD, PhD	Department of Advanced Medical and Surgical Sciences, University of Campania “Luigi Vanvitelli,” Naples, Italy	Drafting/revision of the manuscript for content, including medical writing for content; major role in the acquisition of data
Claudio Gasperini, MD, PhD	Department of Neurosciences, San Camillo-Forlanini Hospital, Rome, Italy	Drafting/revision of the manuscript for content, including medical writing for content; major role in the acquisition of data
Gabriel Gonzalez-Escamilla, MD	Department of Neurology, Focus Program Translational Neuroscience (FTN) and Immunotherapy (FZI), Rhine Main Neuroscience Network (rmn2), University Medical Center of the Johannes Gutenberg University Mainz, Germany	Drafting/revision of the manuscript for content, including medical writing for content; major role in the acquisition of data
Cristina Granziera, MD, PhD	Translational Imaging in Neurology (ThINK) Basel, Department of Biomedical Engineering, Faculty of Medicine, and Neurologic Clinic and Policlinic, MS Center and Research Center for Clinical Neuroimmunology and Neuroscience Basel (RC2NB), University Hospital Basel, University of Basel, Switzerland	Drafting/revision of the manuscript for content, including medical writing for content; major role in the acquisition of data
Sergiu Groppa, MD, PhD	Department of Neurology, Focus Program Translational Neuroscience (FTN) and Immunotherapy (FZI), Rhine Main Neuroscience Network (rmn2), University Medical Center of the Johannes Gutenberg University Mainz, Germany	Drafting/revision of the manuscript for content, including medical writing for content; major role in the acquisition of data
Yael Hacohen, MD, PhD	Queen Square Multiple Sclerosis Centre, Department of Neuroinflammation, UCL Queen Square Institute of Neurology, University College London, United Kingdom	Drafting/revision of the manuscript for content, including medical writing for content; major role in the acquisition of data
Hanne F.F. Harbo, MD, PhD	Department of Neurology, Oslo University Hospital; Institute of Clinical Medicine, University of Oslo, Norway	Drafting/revision of the manuscript for content, including medical writing for content; major role in the acquisition of data
Anna He, MD, PhD	Queen Square Multiple Sclerosis Centre, Department of Neuroinflammation, UCL Queen Square Institute of Neurology, University College London, United Kingdom	Drafting/revision of the manuscript for content, including medical writing for content; major role in the acquisition of data
Einar A. Hogestol, MD, PhD	Department of Neurology, Oslo University Hospital; Institute of Clinical Medicine, and Department of Psychology, University of Oslo, Norway	Drafting/revision of the manuscript for content, including medical writing for content; major role in the acquisition of data
Jens Kuhle, MD, PhD	Neurologic Clinic and Policlinic, MS Center and Research Center for Clinical Neuroimmunology and Neuroscience Basel (RC2NB), University Hospital Basel, University of Basel, Switzerland	Drafting/revision of the manuscript for content, including medical writing for content; major role in the acquisition of data
Sara Llufriu, MD, PhD	Neuroimmunology and Multiple Sclerosis Unit Laboratory of Advanced Imaging in Neuroimmunologic Diseases (ImaginEM), Hospital Clinic Barcelona, Fundació de Recerca Clínic Barcelona-Institut d’Investigacions Biomèdiques August Pi i Su, Spain	Drafting/revision of the manuscript for content, including medical writing for content; major role in the acquisition of data
Carsten Lukas, MD, PhD	Institute of Neuroradiology, and Department of Neurology, St. Josef Hospital, Ruhr-University Bochum, Germany	Drafting/revision of the manuscript for content, including medical writing for content; major role in the acquisition of data
Eloy Martinez-Heras, PhD	Neuroimmunology and Multiple Sclerosis Unit Laboratory of Advanced Imaging in Neuroimmunologic Diseases (ImaginEM), Hospital Clinic Barcelona, Fundació de Recerca Clínic Barcelona-Institut d’Investigacions Biomèdiques August Pi i Su, Spain	Drafting/revision of the manuscript for content, including medical writing for content; major role in the acquisition of data
Silvia Messina, MD, PhD	Nuffield Department of Clinical Neurosciences, University of Oxford, United Kingdom	Drafting/revision of the manuscript for content, including medical writing for content; major role in the acquisition of data
Marcello Moccia, MD, PhD	Department of Molecular Medicine and Medical Biotechnology, University of Naples “Federico II,” Italy	Drafting/revision of the manuscript for content, including medical writing for content; major role in the acquisition of data
Suraya Mohamud, MD	Queen Square Multiple Sclerosis Centre, Department of Neuroinflammation, UCL Queen Square Institute of Neurology, University College London, United Kingdom	Drafting/revision of the manuscript for content, including medical writing for content; major role in the acquisition of data
Riccardo Nistri, MD	Queen Square Multiple Sclerosis Centre, Department of Neuroinflammation, UCL Queen Square Institute of Neurology, University College London, United Kingdom	Drafting/revision of the manuscript for content, including medical writing for content; major role in the acquisition of data
Gro O. Nygaard, MD	Department of Neurology, Oslo University Hospital; Institute of Clinical Medicine, University of Oslo, Norway	Drafting/revision of the manuscript for content, including medical writing for content; major role in the acquisition of data
Jacqueline Palace, MD, PhD	Nuffield Department of Clinical Neurosciences, University of Oxford, United Kingdom	Drafting/revision of the manuscript for content, including medical writing for content; major role in the acquisition of data
Maria Petracca, MD, PhD	Department of Neurosciences and Reproductive and Odontostomatological Sciences, University of Naples “Federico II”; Department of Human Neurosciences, Sapienza University of Rome, Italy	Drafting/revision of the manuscript for content, including medical writing for content; major role in the acquisition of data
Daniela Pinter, PhD	Department of Neurology, Medical University of Graz, Austria	Drafting/revision of the manuscript for content, including medical writing for content; major role in the acquisition of data
Maria A. Rocca, MD, PhD	Neuroimaging Research Unit, Division of Neuroscience, IRCCS San Raffaele Scientific Institute, Neurology Unit, Neurorehabilitation Unit, Neurophysiology Service, IRCCS San Raffaele Scientific Institute; Vita-Salute San Raffaele University, Milan, Italy	Drafting/revision of the manuscript for content, including medical writing for content; major role in the acquisition of data
Alex Rovira, MD	Section of Neuroradiology, Department of Radiology, Hospital Universitari Vall d’Hebron, Universitat Autònoma de Barcelona, Spain	Drafting/revision of the manuscript for content, including medical writing for content; major role in the acquisition of data
Serena Ruggieri, MD, PhD	Department of Neurosciences, San Camillo-Forlanini Hospital, Rome, Italy	Drafting/revision of the manuscript for content, including medical writing for content; major role in the acquisition of data
Jaume Sastre-Garriga, MD, PhD	Centre d’Esclerosi Múltiple de Catalunya (Cemcat), Department of Neurology/Neuroimmunology, Hospital Universitari Vall d’Hebron, Universitat Autònoma de Barcelona, Spain	Drafting/revision of the manuscript for content, including medical writing for content; major role in the acquisition of data
Eva M. Strijbis, MD, PhD	MS Center Amsterdam, Neurology, Vrije Universiteit Amsterdam, Amsterdam Neuroscience, Amsterdam UMC location VUmc, the Netherlands	Drafting/revision of the manuscript for content, including medical writing for content; major role in the acquisition of data
Ahmed T. Toosy, MD, PhD	Queen Square Multiple Sclerosis Centre, Department of Neuroinflammation, UCL Queen Square Institute of Neurology, University College London, United Kingdom	Drafting/revision of the manuscript for content, including medical writing for content; major role in the acquisition of data
Tomas Uher, MD, PhD	Department of Neurology and Center of Clinical Neuroscience, First Faculty of Medicine, Charles University and General University Hospital, Prague, Czech Republic	Drafting/revision of the manuscript for content, including medical writing for content; major role in the acquisition of data
Paola Valsasina, PhD	Neuroimaging Research Unit, Division of Neuroscience, IRCCS San Raffaele Scientific Institute, Neurology Unit, Neurorehabilitation Unit, Neurophysiology Service, IRCCS San Raffaele Scientific Institute; Vita-Salute San Raffaele University, Milan, Italy	Drafting/revision of the manuscript for content, including medical writing for content; major role in the acquisition of data
Manuela Vaneckova, MD, PhD	Department of Radiology, First Faculty of Medicine, Charles University and General University Hospital, Prague, Czech Republic	Drafting/revision of the manuscript for content, including medical writing for content; major role in the acquisition of data
Hugo Vrenken, PhD	MS Center Amsterdam, Radiology and Nuclear Medicine, Vrije Universiteit Amsterdam, Amsterdam Neuroscience, Amsterdam UMC location VUmc, the Netherlands	Drafting/revision of the manuscript for content, including medical writing for content; analysis or interpretation of data
Jed Wingrove, PhD	Queen Square Multiple Sclerosis Centre, Department of Neuroinflammation, UCL Queen Square Institute of Neurology, University College London, United Kingdom	Drafting/revision of the manuscript for content, including medical writing for content; major role in the acquisition of data
Charmaine Yam, MD	Queen Square Multiple Sclerosis Centre, Department of Neuroinflammation, UCL Queen Square Institute of Neurology, University College London, United Kingdom	Drafting/revision of the manuscript for content, including medical writing for content; major role in the acquisition of data
Menno M. Schoonheim, PhD	MS Center Amsterdam, Anatomy and Neurosciences, Vrije Universiteit Amsterdam, Amsterdam Neuroscience, Amsterdam UMC location VUmc, the Netherlands	Drafting/revision of the manuscript for content, including medical writing for content; major role in the acquisition of data
Olga Ciccarelli, MD, PhD	Queen Square Multiple Sclerosis Centre, Department of Neuroinflammation, UCL Queen Square Institute of Neurology, University College London, United Kingdom	Drafting/revision of the manuscript for content, including medical writing for content; major role in the acquisition of data
James H. Cole, PhD	Centre for Medical Image Computing, Department of Computer Science, and Dementia Research Centre, UCL Queen Square Institute of Neurology, University College London, United Kingdom	Drafting/revision of the manuscript for content, including medical writing for content; study concept or design; analysis or interpretation of data
Frederik Barkhof, MD, PhD	Queen Square Multiple Sclerosis Centre, Department of Neuroinflammation, UCL Queen Square Institute of Neurology, University College London, United Kingdom; MS Center Amsterdam, Radiology and Nuclear Medicine, Vrije Universiteit Amsterdam, Amsterdam Neuroscience, Amsterdam UMC location VUmc, the Netherlands; Centre for Medical Image Computing, Department of Medical Physics and Biomedical Engineering, and Dementia Research Centre, UCL Queen Square Institute of Neurology, University College London, United Kingdom	Drafting/revision of the manuscript for content, including medical writing for content; study concept or design; analysis or interpretation of data

## Appendix 2 Coinvestigators

**Table:**

Name	Location	Role	Contribution
Ludwig Kappos, MD, PhD	University Hospital Basel, University of Basel, Switzerland	Site investigator	Coordinated imaging for the site
Tarek Yousry, MD, PhD	UCL Queen Square Institute of Neurology, University College London, United Kingdom	Site investigator	Coordinated imaging for the site

## Glossary

BAG: brain-age gap
DD: disease duration
EDSS: Expanded Disability Status Scale
FLAIR: fluid-attenuated inversion recovery
HI: healthy individuals
MAE: mean absolute error
MSSS: Multiple Sclerosis Severity Score
PwMS: patients with multiple sclerosis
T1w: T1-weighted
